# Fusion-Triggered Switching of Enzymatic Activity on an Artificial Cell Membrane

**DOI:** 10.3390/s120505966

**Published:** 2012-05-09

**Authors:** Masaru Mukai, Yoshihiro Sasaki, Jun-ichi Kikuchi

**Affiliations:** 1 Graduate School of Materials Science, Nara Institute of Science and Technology, 8916-5 Takayama, Ikoma, Nara 630-0192, Japan; 2 Nanotube Research Center, National Institute of Advanced Industrial Science and Technology, Tsukuba Central 5, 1-1-1 Higashi, Tsukuba, Ibaraki 305-8565, Japan; E-Mail: mukai-m@aist.go.jp; 3 Institute of Biomaterials and Bioengineering, Tokyo Medical and Dental University, 2-3-10 Kanda-Surugadai, Chiyoda, Tokyo 101-0062, Japan; E-Mail: ysasaki.org@tmd.ac.jp; 4 PRESTO, Japan Science and Technology Agency, 4-1-8 Honcho Kawaguchi, Saitama 332-0012, Japan

**Keywords:** liposome, enzyme, liposome fusion, self-assembly, phase transition, molecular device

## Abstract

A nanosensory membrane device was constructed for detecting liposome fusion through changes in an enzymatic activity. Inspired by a biological signal transduction system, the device design involved functionalized liposomal membranes prepared by self-assembly of the following molecular components: a synthetic peptide lipid and a phospholipid as matrix membrane components, a Schiff's base of pyridoxal 5′-phosphate with phosphatidylethanolamine as a thermo-responsive artificial receptor, NADH-dependent *L*-lactate dehydrogenase as a signal amplifier, and Cu^2+^ ion as a signal mediator between the receptor and enzyme. The enzymatic activity of the membrane device was adjustable by changing the matrix lipid composition, reflecting the thermotropic phase transition behavior of the lipid membranes, which in turn controlled receptor binding affinity toward the enzyme-inhibiting mediator species. When an effective fusogen anionic polymer was added to these cationic liposomes, membrane fusion occurred, and the functionalized liposomal membranes responded with changes in enzymatic activity, thus serving as an effective nanosensory device for liposome fusion detection.

## 1. Introduction

**Figure 1.**
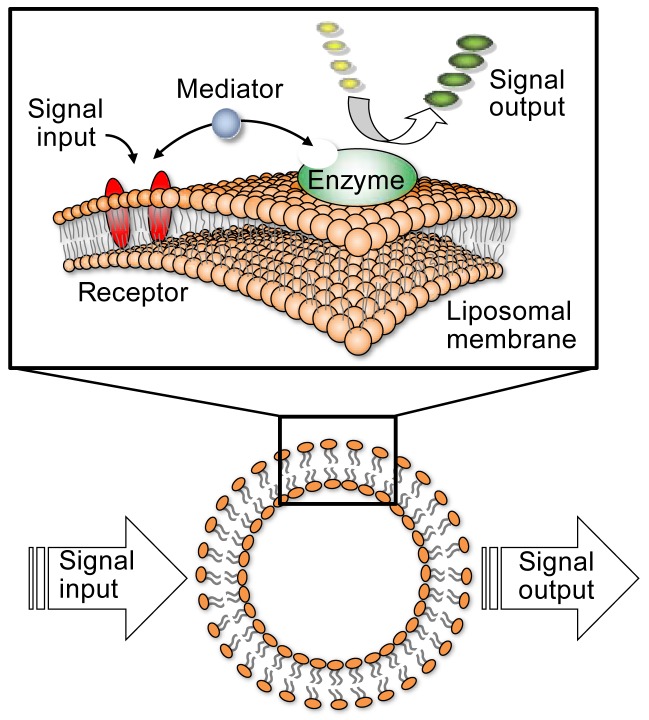
Schematic illustration of a liposomal molecular device inspired by biological signal transduction system.

Recently, much effort has been devoted to developing nanoscale devices using molecules or molecular devices composed of molecular elements, such as switches, wires, and logic gates, and capable of extending current semiconductor technology to nanoscale information technology [1–4]. However, integration of these functional elements to produce real molecular devices still remains a challenge. A biologically inspired approach may present a unique solution for achieving integrated system architectures that will orchestrate a huge number of molecular devices inside future nanomachines. In this respect, our recent attention has been focused on functional simulation of biological signal transduction systems by employing self-organized molecular assemblies in aqueous media. A signal transduction system located in the cell membrane is an example of naturally occurring nanodevices, in which signal transmission among functional biomolecules, such as receptors and enzymes, is efficiently achieved in the cell membrane [5]. Previously, we have reported on artificial cell membrane-type nanodevices, employing a concept inspired by biological signal transduction, which entails a system essentially comprised of three molecular components: a synthetic receptor, enzyme, and liposomal membrane (Figure 1). The receptor and the enzyme are self-assembled on the liposomal membrane through noncovalent interactions and the enzyme's catalytic activity is controlled by an external signal subsequent to receptor activation, with signal transduction mediated by metal ions. In this system, the receptor adjusts the enzymatic activity depending on the molecular recognition for a specific signal [6–16].

**Figure 2.**
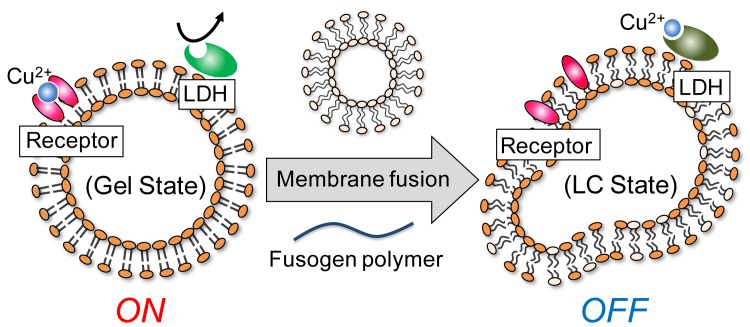
Schematic illustration of a bio-inspired molecular device that detects liposome fusion by altering the activity of an enzymatic reaction. Left and right figures represent on and off-states of *L*-lactate dehydrogenase (LDH) before and after membrane fusion, respectively. The lipid bilayers are in gel and liquid-crystalline (LC) phases for on and off-states, respectively.

**Figure 3.**
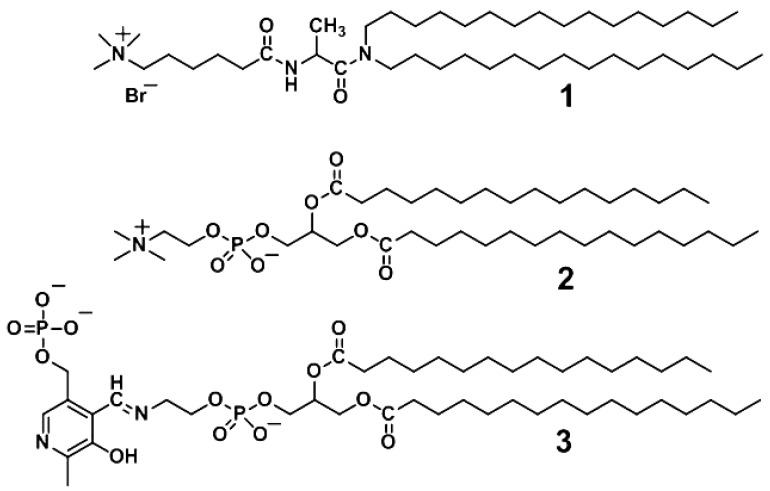
Molecular components of the bio-inspired molecular device.

This paper reports the construction of a bio-inspired molecular device that senses membrane fusion by changes in membrane-bound enzyme activity. Membrane fusion is one of the most fundamental processes in biological system, involved in cargo transport through secretory pathways, fertilization, organelle inheritance, and viral entry into host cells [17–22], but there have been few reports of a molecular device sensing membrane fusion. The present system functions through cooperation of a thermo-responsive receptor and a natural enzyme, with a signal mediator, as a means of converting a liposomal membrane state change into a measurable enzyme response (Figure 2). The liposomal platform was constructed with an incorporated cationic peptide lipid (**1**), a phospholipid (**2**), and three functional elements: a Schiff's base of pyridoxal 5′-phosphate (PLP) with phosphatidylethanolamine (**3**) as a thermo-responsive artificial receptor; NADH-dependent *L*-lactate dehydrogenase (LDH) as an effector; and copper (II) (Cu^2+^) ions as the signal mediator (Figure 3). In this study, we report an examination of this system's enzymatic activity in response to various conditions and additives, adjustment of the system's lipid composition while monitoring the phase transition temperature, and detection by the designed enzymatic response of phase transitions triggered by liposome fusion.

The strategy for the design of this molecular device involved taking advantage of specific attributes of the three incorporated components. Receptor **3**'s ability to change its binding affinity toward metal ions, depending on the liposomal membrane's phase state [13] was one of the receptor's most important properties in the present fusion sensing system. When the liposome is in a gel state, the receptor has higher binding affinity for the signal mediator than does the enzyme, which results in an enzymatically active state, or “on state” (Figure 2). It is well known that the liposome fusion can be induced by a membrane-interacting polymer as a fusogen. For example, the fusion of cationic liposomes was induced by anionic polymers [23,24]. When liposome fusion occurs in the presence of liposomes functionalized with both receptor and enzyme, the maxtrix lipid composition shifts during fusion, changing the liposome's phase state from gel to liquid-crystal. We have previously reported here that a similar supramolecular system using receptor **3** and LDH markedly decreases enzymatic activity within a specific temperature range, reflecting the membrane's gel to liquid-crystalline phase transition [14]. This is due to increased mediator binding to the enzyme as a result of decreased mediator binding affinity toward the receptor. As the Cu^2+^ ions are an LDH competitive inhibitor, this molecular device turns the system to the enzymatically inactive state, or “off state” (Figure 2). Thus, the present membrane device detects liposome fusion by translating the state change to an enzymatic response.

## 2. Experimental Section

### 2.1. Materials

*N,N*-Dihexadecyl-*N*^α^-(trimethylammonio)hexanoyl-*l*-alaninamide bromide (**1**) was prepared as previously described [25]. The following compounds were commercially available and used without further purification: 1,2-dipalmitoyl-*sn*-glycero-3-phosphocholine (**2**, NOF Corporation, Tokyo, Japan), 1,2-dimyristoyl-*sn*-glycero-3-phosphoethanolamine (DMPE, Avanti Polar Lipids, Inc., Alabaster, AL, USA), pyridoxal 5′-phosphate (PLP, Sigma-Aldrich, St. Louis, MO, USA), *l*-lactate dehydrogenase (LDH) from pig heart (Roche Diagnostics GmbH, Basel, Switzerland), *β*-nicotinamide adenine dinucleotide disodium salt (NADH, Sigma-Aldrich), sodium pyruvate (Wako Pure Chemical Industries, Ltd., Osaka, Japan), copper(II) perchlorate hexahydrate (Kanto Chemical Co., Inc., Tokyo, Japan), *N*-(7-nitrobenz-2-oxa-1,3-diazol-4-yl)-1,2-dihexadecanoyl-*sn*-glycero-3-phosphoethanolamine (NBD-PE, Invitrogen, Life Technologies, Grand Island, NY, USA), Lissamine Rhodamine B 1,2-dihexadecanoyl-*sn*-glycero-3-phosphoethanolamine (Rh-PE) (Invitrogen), poly(vinyl alcohol) and poly(viny1 sulfate) potassium salt (PVA and PVSK, respectively, Wako Pure Chemical Industries). Other chemicals were of analytical grade.

### 2.2. Preparation of Liposomes

Giant liposomes were prepared using an established protocol [26]. Briefly, appropriate amounts of lipids **1** and **2** and DMPE were dissolved in chloroform, the solvent evaporated under a nitrogen gas stream, and residual trace solvent completely removed *in vacuo*. Hydration of the resulting thin film on the vial wall was performed at 50 °C with an appropriate amount of pure water or 2-[4-(2-hydroxyethyl)-1-piperazinyl]ethanesulfonate (HEPES) buffer (10 mM, pH 7.0) to produce DMPE and binary lipid (**1** and **2**) concentrations of 0.050 and 1.0 mM, respectively. Multilamellar liposomes with a 200–300 nm diameter were formed by vortex mixing the aqueous dispersion of the thin lipid film. Small unilamellar liposomes with a 60 nm diameter were prepared by sonication of the multilamellar liposomes using a cup-type sonicator (Sonifier 250D, Branson Ultrasonics Corp., Danbury, CT, USA) at 30 W for 20 min and above the phase transition temperature. Receptor **3** was prepared by addition of PLP to liposomes containing DMPE and incorporation confirmed by electronic absorption spectra [9,13].

### 2.3. Measurements

Electronic absorption spectra were recorded on a Shimadzu UV-2400 spectrophotometer (Shimadzu, Kyoto, Japan). Fluorescence spectra were collected with a Hitachi F-4500 spectrofluorometer (Hitachi High-Technologies Corp., Tokyo, Japan). Differential scanning calorimetry (DSC) was performed on an ultrasensitive microcalorimeter (VP-DSC; MicroCal Software Inc., GE Healthcare Japan Corp., Tokyo, Japan). Fluorescence microscopic observation was carried out using an Olympus IX71 microscope with the images recorded using an Olympus DP70 color CCD camera (Olympus, Center Valley, PA, USA).

### 2.4. Enzyme Assay

LDH activity was evaluated in HEPES buffer (10 mM, pH 7.0) at 35 °C using sodium pyruvate as a substrate. A 1 mL sample solution was prepared by mixing LDH and NADH (2.8 nM and 0.25 mM, respectively) in the presence or absence of an appropriate amount of Cu^2+^ ions and liposomes, and the reaction initiated by addition of pyruvate (0.50 mM). Under the enzyme assay conditions, concentrations of LDH, NADH and pyruvate were set to give the maximum initial velocity. The catalytic activity was evaluated spectrophotometrically by measuring NADH consumption coupled with pyruvate reduction to *L*-lactate. The NADH molar extinction coefficient at 340 nm was 6,220 M^−1^·cm^−1^.

## 3. Results and Discussion

### 3.1. Design of Bio-Inspired Molecular Device

Liposomes are spherical, hollow particles with a lipid bilayer shell several nanometers thick and have been frequently used in drug and gene delivery systems and as bioreactors [27–29]. In this study, a liposome formed with a cationic peptide lipid (**1**) and a phospholipid (**2**) was employed as scaffolding for a molecular device designed to sense liposome fusion. A water-soluble enzyme, LDH, was immobilized on the external liposomal surface through electrostatic interactions [30], with LDH catalytic activity retained; in contrast, activity was not retained by LDH on micelles of the cationic surfactant cetyltrimethylammonium bromide, which denatures LDH, extinguishing catalytic activity [31]. Thus, the cationic liposomal membrane used here served as a sophisticated scaffold for the supramolecular assembly of an enzyme and a synthetic receptor.

Some metal ions act as enzyme inhibitors and we have previously reported that Cu^2+^ ions potently inhibit LDH in a reversible and competitive manner, regardless of its residence on liposomes [31].

A functional synthetic receptor in the current system must fulfill the following requirements: (i) the receptor effectively binds to the liposomal membrane; (ii) the receptor provides a molecular recognition site for a metal ion acting as a mediator between the receptor and enzyme; and (iii) the receptor binding affinity toward the mediator varies according to the membranes' lipid composition. Based on these criteria, receptor **3** was employed as it is capable of changing metal ion binding affinity depending on the liposome phase state [13].

### 3.2. Effect of Lipid Matrix on Enzymatic Activity

**Figure 4.**
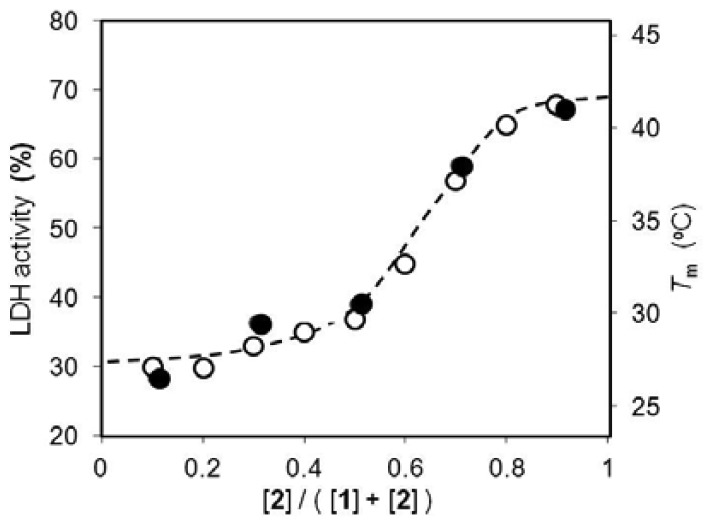
Correlations of lipid molar fraction with LDH activity (open circle) and phase transition temperature (closed) of the bio-inspired molecular device in HEPES buffer (10 mM) at pH 7.0 and 35 °C. Concentrations in mM: [**1**]+[**2**], 1.0; [DMPE], 0.05; [PLP], 0.02; [LDH], 2.8 × 10^−6^; [NADH], 0.25; [pyruvate], 0.50; and [Cu^2+^], 4.0 × 10^−3^.

The effect of the liposome lipid matrix on enzymatic activity was evaluated using multilamellar liposomes formed from lipids **1** and **2** at various molar ratios. The transition temperatures from the gel to liquid-crystalline phase were observed from 25 to 41 °C by DSC (Figure 4). The results indicated that the liposome phase transition was easily controlled by the lipid molar ratio.

The activity of LDH immobilized on the liposome also functionalized with the receptor and mediator was evaluated in HEPES buffer (10 mM, pH 7.0) and at 35 °C. The reduction rate of pyruvate to *l*-lactate was monitored spectrophotometrically by following the concurrent NADH consumption rate (*v*_0_), and the LDH activity represented as the *v*_0_ magnitude in the presence of Cu^2+^ ions relative to the copper-free system. The lipid matrix dependence of LDH activity showed good correlation with the phase transition temperature (Figure 4).

When the molar ratio of lipid **1** to lipid **2** was changed from 5/5 to 2/8, LDH activity drastically switched from 37% to 65%. At 35 °C, the liposomes with a molar ratio of 2/8 and 5/5 were in a gel and liquid-crystalline state, respectively, and thus, the enzymatic activity was clearly capable of switching on and off below and above the membrane's phase transition temperature, respectively.

Receptor **3** has previously been shown to decrease the binding constant for Cu^2+^ ions in the liquid-crystalline state compared to the gel state [13]. In the latter state, receptor **3** exhibits higher affinity for Cu^2+^ ions than the enzyme, resulting in an enzymatically active state. On the other hand, the liquid-crystalline state allows the enzyme to bind Cu^2+^ ions more strongly than the receptor and switches off activity, the Cu^2+^ ion competitively inhibiting LDH. These results indicated that, if the present functionalized liposomes changed their phase state upon liposome fusion, fusion events could be detected by changes in enzymatic activity.

### 3.3. Liposome Fusion Behavior

It is well known that liposome fusion can be induced by water-soluble polymers [23,24]. The present work utilized an anionic and a non-ionic polymer, poly(viny1 sulfate) potassium salt (PVSK) and poly(vinyl alcohol) (PVA), respectively. Fusion efficiency upon addition of these polymers to liposomes was evaluated by a published method using fluorescence resonance energy transfer (FRET) [32]. Briefly, liposome fusion was monitored using a couple of fluorescent phosphatidylethanolamine derivatives containing a rhodamine (Rh-PE) or *N*-(7-nitrobenz-2-oxa-1,3-diazol-4-yl) moiety (NBD-PE). The NBD-PE absorption and fluorescence maxima appeared at 450 and 530 nm, respectively. On the other hand, the Rh-PE has absorption and fluorescence maxima at 540 and 590 nm, respectively. If Rh-PE was located close to NBD-PE in the liposome, NBD-PE excitation resulted in Rh-PE emission at 590 nm due to FRET behavior.

**Figure 5.**
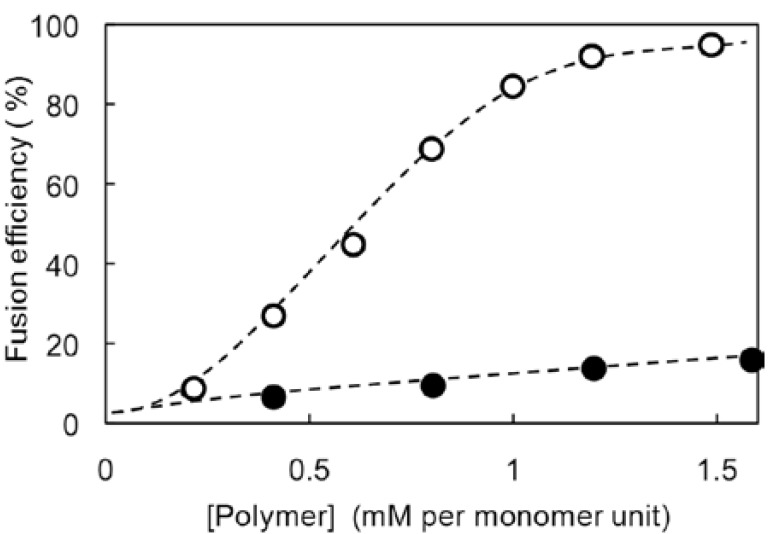
Dependences of polymer concentration on liposome fusion efficiency for PVSK and PVA (open and closed circles, respectively) evaluated by FRET assay. Aqueous fluorescence-labeled liposomes composed of lipids **1** and **2** (0.20 and 0.80 mM, respectively) and Rh-PE and NBD-PE (both 0.50 μM) were mixed with unlabeled aqueous liposomes composed of **1** and **2** (both 0.50 mM) in a volume ratio of 1/9 (v/v) at pH 7.0 and 35 °C; fusion efficiency evaluated for by addition of various concentrations of a polymer.

Fluorescence-labeled liposomes were prepared by sonication of an aqueous liposome dispersion composed of lipids **1** and **2** (0.20 and 0.80 mM, respectively) in the presence of Rh-PE and NBD-PE (both 0.5 μM). The resulting liposomes were mixed with unlabeled aqueous liposomes, composed of **1** and **2** (both 0.50 mM), in a 1/9 (v/v) ratio at pH 7.0 and 35 °C. Upon NBD-PE excitation at 450 nm, the resulting solutions showed fluorescence emission at 530 and 590 nm, reflecting emissions from NBD-PE and Rh-PE, respectively. When PVSK or PVA was added to the aquous liposome, the fluorescence intensity at 530 nm increased as polymer concentration increased, with a concomitant decrease of the intensity at 590 nm. Thus, fusion efficiency was evaluated using a calibration set of fluorescent spectra, allowing description of fusion dependence on polymer concentration (Figure 5). Anionic PVSK was bound on the cationic liposome surface through electrostatic interactions to induce membrane fusion probably due to dehydration and disruption of membrane surface. On the other hand, nonionic PVA showed little effect on fusion of cationic liposomes as compared with anionic PVSK. In the present cationic liposomal system, anionic PVSK was the more effective fusogen compared with non-ionic PVA.

**Figure 6.**
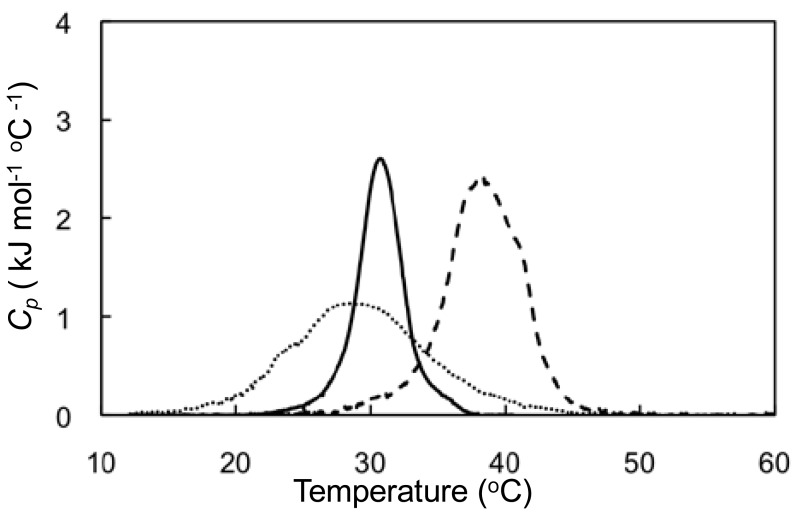
DSC thermograms from aqueous liposomes composed of lipids **1** and **2** at a 5/5 or 2/8 molar ratio (dotted and dashed line, respectively) with no polymer and their 9/1 (v/v) mixture with PVSK (solid line). Concentrations in mM: [**1**]+[**2**], 1.0 and [PVSK], 0.80 (per monomer unit).

Polymer-induced fusion behavior was also evaluated by DSC measurements (Figure 6), and it was found that small unilamellar liposomes composed of lipids **1** and **2** at a 5/5 molar ratio and 60 nm diameter showed a broad phase transition from the gel to liquid-crystalline state, with a maximum at 28.1 °C. On the other hand, liposomes with a molar ratio of 2/8 exhibited a phase transition at 39.5 °C. When 5/5 lipid molar ratio liposomes were mixed those with a 2/8 molar ratio in a 9/1 (v/v) ratio, the DSC thermogram was a simple overlay of the individual DCS thermograms. Upon addition of PVSK to the mixed liposome preparation, the thermogram changed to show a single phase transition with a maximum at 30.8 °C, which was in good agreement with thermograms for large unilamellar liposomes composed of lipids **1** and **2** at a 47/53 molar ratio and a maximum at 31.4 °C, corresponding to liposomes formed through ideal fusion.

**Figure 7.**
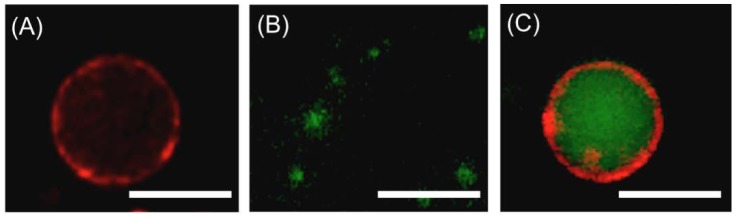
Fluorescence microscopic images of a giant liposome composed of **1** and **2** at a 2/8 molar ratio and labeled with Rh-PE (**A**), small unilamellar liposomes at a 5/5 molar ratio and labeled with rhodamine 123 (**B**), and their 1/9 (v/v) mixture with PVSK (**C**). Concentrations in mM: [**1**]+[**2**], 1.0 and [PVSK], 0.80 (per monomer unit); scale bar, 10 μm.

PVSK-induced fusion of liposomes was also observed by fluorescence microscopy. Giant liposomes composed of lipids **1** and **2** at a 2/8 molar ratio and ca. 10 μm were prepared and the membranes labeled with red fluorescent Rh-PE (Figure 7(A)). Small unilamellar liposomes at a 5/5 lipid molar ratio and 60 nm were also prepared and the inner aqueous phase labeled with green fluorescent rhodamine 123 (Figure 7(B)). The aqueous dispersions of giant and small unilamellar liposomes were then mixed in a 1/9 (v/v) ratio, followed by PVSK addition. The resulting giant liposomes possessed clearly observed red-colored lipid bilayers and green-colored inner aqueous phases, indicating that PVSK induced liposome fusion (Figure 7(C)).

### 3.4. Switching of Enzymatic Activity

Based on the above results, the performance of these functionalized liposomes as molecular devices for sensing liposome fusion was next kinetically evaluated by means of electronic absorption spectroscopy. Enzymatic activity was evaluated as the initial velocity (*v*_0_) magnitude in the presence of Cu^2+^ ions relative to the absence of Cu^2+^ ions.

In homogeneous aqueous solution, LDH activity was reduced to 4% of the Cu^2+^-free system by 4.0 μM Cu^2+^ ions (Table 1, Entries 1 and 2). Upon addition of polymers, such as PVA or PVSK, the enzymatic activity was comparable to the polymer-free system (Table 1, Entries 3 and 5), indicating that these polymers did not influence enzymatic activity. The enzyme inhibition response to Cu^2+^ ions in the presence of polymers was also analogous to the polymer-free system (Table 1, Entries 4 and 6).

In a liposome system composed of lipids **1** and **2** at a 2/8 molar ratio containing the receptor **3**, LDH relative activity in the presence of Cu^2+^ ions was 68% of the Cu^2+^-free system (Table 1, Entries 7 and 8). The higher enzymatic activity in liposome system than that without liposome in the presence of Cu^2+^ ions strongly suggests that the pyridoxal moiety of receptor **3** acted as an effective metal-binding site. That is, as the liposomes were in a gel state under the present conditions, the receptor **3** binding affinity for Cu^2+^ ions was much higher than the enzyme and, thus, LDH activity drastically recovered in the functionalized liposome compared to the homogeneous aqueous solution.

In the mixed liposomal system containing the liposomes composed of lipids **1** and **2** at both 2/8 and 5/5 molar ratios, LDH activity in the presence of Cu^2+^ ions and PVA was 62% of the Cu^2+^-free system (Table 1, Entries 9 and 10). The Cu^2+^-inhibition effect was comparable to the corresponding polymer-free system (Table 1, Entries 7 and 8), as PVA scarcely induced liposome fusion. On the other hand, in the mixed liposome system containing PVSK instead of PVA, LDH activity in the presence of Cu^2+^ ions was 42% of the Cu^2+^-free system (Table 1, Entries 11 and 12). The difference in LDH activities in the presence of Cu^2+^ ions upon addition of either polymer clearly reflected the difference in their fusion efficiencies. At 35°C, liposomes composed of lipids **1** and **2** at 2/8 and 5/5 molar ratios are in a gel and liquid-crystalline state, respectively. When PVSK induced fusion of these functionalized liposomes, the fused liposomes transitioned from the gel to liquid-crystalline state. Thus, receptor binding affinity toward the mediator decreased, enhancing mediator inhibition of the enzyme.

## 4. Conclusions

A novel bio-inspired molecular device was created in which enzymatic activity on a liposomal membrane was switched on or off by a thermo-responsive receptor sensitive to changes in the phase state of membrane. Although the switching range of the present system should be expanded much more, the present supramolecular system represents a unique signal transduction behavior, detecting liposome fusion through its response in enzyme activity changes. Further improvement of the present sensing system will be achieved, for example, by development of a potent artificial receptor exhibiting more drastic switching behavior for the recognition of mediator species above and below the phase transition of lipid membrane. Attempts to apply this system to molecular communication are currently underway [33], representing a new communication paradigm by using molecules as information carriers.

## Figures and Tables

**Table 1.** Enzymatic activity on liposomes in HEPES buffer (10 mM) at pH 7.0 and 35 °C ^a^.

**Entry**	**Component**			***v _0_*** **(nM·s^−1^)** ^b^	**Activity** **(%)** ^c^

	**Liposome**	**Polymer**	**Metalion**		
1	none	none	none	360	100
2	none	none	Cu^2+^	10	4
3	none	PVA	none	340	100
4	none	PVA	Cu^2+^	20	6
5	none	PVSK	none	340	100
6	none	PVSK	Cu^2+^	20	6
7	**1** & **2** (2/8)^d^	none	none	290	100
8	**1** & **2** (2/8)^d^	none	Cu^2+^	200	68
9	**1** & **2** (mix)^e^	PVA	none	290	100
10	**1** & **2** (mix)^e^	PVA	Cu^2+^	180	62
11	**1** & **2** (mix)^e^	PVSK	none	290	100
12	**1** & **2** (mix)^e^	PVSK	Cu^2+^	120	42

a Concentrations in mM: [**1**]+[**2**], 1.0; [DMPE], 0.05; [PLP], 0.02; [LDH], 2.8 × 10^−6^; [NADH], 0.25; [pyruvate], 0.5; [Cu^2+^], 4.0 × 10^−3^; and [polymer], 0.8;

b Initial velocity for LDH reduction of pyruvate to *l*-lactate monitored by spectrophotometric detection of NADH consumption; values accurate within ±5%;

c Magnitude of *v*_0_ in presence of Cu^2+^ ions compared to that in the Cu^2+^-free system;

d Liposome composed of **1** and **2** in 2/8 molar ratio and receptor;

e 1/9 (v/v) Mixture of aqueous liposome composed of **1** and **2** (molar ratio, 2/8) with receptor and aqueous liposome composed of **1** and **2** (molar ratio, 5/5) without receptor.
